# Supplementary material for: Combined deletion of Glut1 and Glut3 impairs lung adenocarcinoma growth
Source: eLife. 2020 Jun 23;9:e53618. doi: 10.7554/eLife.53618 (PMC7311173; doi:10.7554/eLife.53618)
Supplement: Transparent reporting form [file elife-53618-transrepform.docx]

***eLife’s* transparent reporting form**

We encourage authors to provide detailed information *within their submission* to facilitate the interpretation and replication of experiments. Authors can upload supporting documentation to indicate the use of appropriate reporting guidelines for health-related research (see [EQUATOR Network](http://www.equator-network.org/%20)), life science research (see the [BioSharing Information Resource](https://biosharing.org/)), or the [ARRIVE guidelines](http://www.plosbiology.org/article/info:doi/10.1371/journal.pbio.1000412) for reporting work involving animal research. Where applicable, authors should refer to any relevant reporting standards documents in this form.

If you have any questions, please consult our Journal Policies and/or contact us: [editorial@elifesciences.org](mailto:editorial@elifesciences.org).

**Sample-size estimation**

- You should state whether an appropriate sample size was computed when the study was being designed
- You should state the statistical method of sample size computation and any required assumptions
- If no explicit power analysis was used, you should describe how you decided what sample (replicate) size (number) to use

Please outline where this information can be found within the submission (e.g., sections or figure legends), or explain why this information doesn’t apply to your submission:

The sample size (n) and the statistical method are indicated in the figure, in the figure legend, or in the Materials and Methods section.

No power analyze was applied. Knowing that a heterogeneity exists between mice, cohorts of 4 or more animals per condition were used.

**Replicates**

- You should report how often each experiment was performed
- You should include a definition of biological versus technical replication
- The data obtained should be provided and sufficient information should be provided to indicate the number of independent biological and/or technical replicates
- If you encountered any outliers, you should describe how these were handled
- Criteria for exclusion/inclusion of data should be clearly stated
- High-throughput sequence data should be uploaded before submission, with a private link for reviewers provided (these are available from both GEO and ArrayExpress)

Please outline where this information can be found within the submission (e.g., sections or figure legends), or explain why this information doesn’t apply to your submission:

Information about biological and technical replicates are indicated in the figure legends, as well as in the Materials and Methods section.

For the μCT analyses, some tumors could not be followed because tumor advancement led sometimes to tumor fusion, avoiding individual tumor volume follow-up. Consequently, we did not consider these fused tumors.

The RNA sequencing data have been deposited to the GEO database (<https://www.ncbi.nlm.nih.gov/geo/>) and assigned the identifier: GSE138757. Password for reviewers: uxmtogmortsxzsx

**Statistical reporting**

- Statistical analysis methods should be described and justified
- Raw data should be presented in figures whenever informative to do so (typically when N per group is less than 10)
- For each experiment, you should identify the statistical tests used, exact values of N, definitions of center, methods of multiple test correction, and dispersion and precision measures (e.g., mean, median, SD, SEM, confidence intervals; and, for the major substantive results, a measure of effect size (e.g., Pearson's r, Cohen's d)
- Report exact p-values wherever possible alongside the summary statistics and 95% confidence intervals. These should be reported for all key questions and not only when the p-value is less than 0.05.

Please outline where this information can be found within the submission (e.g., sections or figure legends), or explain why this information doesn’t apply to your submission:

For each experiment, all data related to statistical reporting are indicated in the figure legend or in the Materials and Methods section. Except when p-values were inferior to 0.0001 (****) or superior to 0.9999 (ns: not significant), we showed the exact p-values on graphs.

(For large datasets, or papers with a very large number of statistical tests, you may upload a single table file with tests, Ns, etc., with reference to sections in the manuscript.)

**Group allocation**

- Indicate how samples were allocated into experimental groups (in the case of clinical studies, please specify allocation to treatment method); if randomization was used, please also state if restricted randomization was applied
- Indicate if masking was used during group allocation, data collection and/or data analysis

Please outline where this information can be found within the submission (e.g., sections or figure legends), or explain why this information doesn’t apply to your submission:

Group allocation are described in the Material and Methods section. We could, furthermore, specify that groups of mice were defined either on Glut1, Glut3 or Glut1 and Glut3 genotype(s). Concerning the PPARαΔN experiment, we did a μCT scan of each mouse prior doxycycline addition and split them into 4 groups (KP, KPG1, KP_PPARαΔN (doxycycline induction), KPG1_PPARαΔN (doxycycline induction)) containing mice with comparable tumor advancement between KP - KP_PPARαΔN and between KPG1 - KPG1_PPARαΔN.

**Additional data files (“source data”)**

- We encourage you to upload relevant additional data files, such as numerical data that are represented as a graph in a figure, or as a summary table
- Where provided, these should be in the most useful format, and they can be uploaded as “Source data” files linked to a main figure or table
- Include model definition files including the full list of parameters used
- Include code used for data analysis (e.g., R, MatLab)
- Avoid stating that data files are “available upon request”

Please indicate the figures or tables for which source data files have been provided:

The figures for which source data files have been provided are the following:

- **Figure 1**: Figure 1d, Figure 1g, Figure 1h
- **Figure 1 – figure supplement 2**: Figure 1 – figure supplement 2a
- **Figure 3**: Figure 3a
- **Figure 3 – figure supplement 1**: Figure 3 – figure supplement 1a
- **Figure 3 – figure supplement 3**: Figure 3 – figure supplement 3c
- **Figure 4**: Figure 4c, Figure 4f, Figure 4g
- **Figure 4 – figure supplement 2**: Figure 4 – figure supplement 2a, Figure 4 – figure supplement 2b, Figure 4 – figure supplement 2c, Figure 4 – figure supplement 2d
- **Figure 4 – figure supplement 3**: Figure 4 – figure supplement 3a
